# ‘Social Re-presentation for…’: An Action-Oriented Formula for Intergroup Relations Research

**DOI:** 10.3389/fpsyg.2020.00352

**Published:** 2020-03-03

**Authors:** Luke J. Buhagiar, Gordon Sammut

**Affiliations:** ^1^Department of Psychology, University of Malta, Msida, Malta; ^2^Department of Criminology, University of Malta, Msida, Malta

**Keywords:** social representations, project, collective action, intergroup relations, alternative representations, joint intentionality, social influence, conflict

## Abstract

Intergroup relations are of crucial importance in contemporary times, with concerns around social representations, social influence and collective action remaining salient. A core aspect of intergroup conflict revolves around the notion of joint projects, whereby different collectives seek to promote their own project through processes of joint intentionality. Nonetheless, we contend that intergroup relations research can tackle the notion of projects more fruitfully by studying the mutual understandings of projects of groups in conflict. Accordingly, we propose an action-oriented reformulation for intergroup relations research, which is contrasted with the standard object-oriented formula. Object-oriented research either (a) emphasizes the study of social objects without regard for their different construal by members of conflicting groups, or (b) focuses on ‘social representations of’ the objects in question, without regard for the projects that such representations serve. Contrastingly, action-oriented research (a) seeks to understand a collective’s ‘social re-presentation *for*’ a specific project; and (b) studies the social and alternative re-presentation of objects and projects as a systemic product of intergroup relations. We then present illustrative examples of object-oriented research, followed by a study concerning Arab-Maltese relations in Malta as an example of action-oriented research. We end by making recommendations for future research on intergroup relations, with the aim of shedding light on the processes that bind coalitions for collective action.

## Introduction: Groups in Conflict

Intergroup relations remain a prevalent concern in many countries in Europe and beyond. Populist and other movements contest regional and national definitions of identity as well as institutional establishments. Similar contestations are evident as definitions of climate change, fair trade, equality, political interference, and terrorism are contested around the world. Groups in conflict build social psychological repertoires involving degrees of closed-mindedness and other cognitive biases in the service of their aims (Bar-Tal, 2011, p. 11). Such repertoires do not simply include attitudes and perceptions linked directly to the conflict, but also others carried over from contexts prior to the conflict itself (Bar-Tal, 2011, p. 4). In the process, self-serving social psychological processes (Ross and Nisbett, 2011) – such as naive realism (Ross and Ward, 1996) and the confirmation bias (Nickerson, 1998) – can potentially result in different construals (Ross and Ward, 1996) of the same contested object. Groups in conflict often resist the view that their outgroup’s actions are based on different understandings of mutually relevant information (Robinson et al., 1995). Rather, they assume that the outgroup’s views are biased, misinformed, or downright ignorant (Ross and Ward, 1996; Sammut and Sartawi, 2012). This promotes further inflexibility in viewpoints, often fueling spirals of conflict between groups (Sammut et al., 2015). Perceptions are thus a key concern in intergroup conflict (Fisher and Kelman, 2011, p. 61), precisely because without understanding perceptions it remains unclear what the mutual concerns and attributions of both sides are. Indeed, perceptions also feature centrally in contact and prejudice research, which studies the possibility of conflict attenuation by means of specified forms of intergroup contact (Dixon et al., 2015). Such studies, and related studies of intergroup threats as they unfold over spatial and temporal dimensions, have been a cornerstone of research aimed at conflict resolution (Dixon et al., 2019).

Alongside mainstream research, social representations theory (SRT) has provided a useful framework for studying intergroup relations. At the heart of SRT lies the view that “powerful majorities attempt to define the meaning of new or otherwise important information as a function of their group norms, while subordinate minorities employ propaganda techniques of social influence to resist majority influence and propose alternative positions” (Staerklé et al., 2011, p. 759). Whilst various definitions of social representations abound in the literature, the systemic and functional aspects of social representations transpire as core features. Sammut and Howarth (2014) define social representations as “*systems* of communication and social *influence* that constitute the social realities of different groups in society [emphasis added]” (p. 1799–1800).

In this paper, we contend that intergroup relations research can better tap into the systemic and functional aspects of social representations than it has to date (cf. Wagner, 1998; Sammut et al., 2012; Lopes and Gaskell, 2015). Essentially, the question of how changes in social representations might unfold as a function of minority/majority relations is yet to be addressed (Marková, 2008). The processes by which different groups promote contrasting representations of both (a) contested objects and (b) contested projects, remain understudied in intergroup relations research. We argue that intergroup relations research benefits from an *action-oriented* social representations formula that prioritizes the systemic and functional aspects inherent in contrasting representations. Our proposal entails a conception of social representations as being necessarily *for* joint projects (Bauer and Gaskell, 1999; cf. Franks, 2011). By foregrounding joint projects and collective action, we argue that an action-oriented approach is better suited in addressing key issues involving intergroup dynamics, both within and outside of SRT. We contrast this approach with the prevailing object-oriented view, which gives primacy to descriptions and characterizations of objects being represented. We end by making recommendations for future research. Throughout the paper, we use ‘re-presentation’ to refer to social re-presentation as process, and ‘representation’ to denote a social representation’s content (see Chryssides et al., 2009).

## Intergroup Relations: Construals of Objects and Projects

According to Bar-Tal 2011, (p. 1), intergroup conflicts constitute “situations in which two or more parties *perceive* that their goals and/or interests are in direct contradiction with one another and *decide* to act on the basis of this perception” (Bar-Tal, 2011, p. 1). Such perceptions are underpinned by “universal cognitive and motivational biases that characterize all human beings as they are, in every context” (Bar-Tal and Halperin, 2011, p. 228). Chief among these are naïve realism (Ross and Ward, 1996) and motivated reasoning (Kunda, 1990; Molden and Higgins, 2005). Naïve realism constitutes the belief that one sees things objectively, and that others will have the same views if they process the same information in an unbiased manner (Ross and Ward, 1996, p. 110–111). Such beliefs are appreciable in the false consensus effect, where individuals overestimate the popularity of their choices (Ross et al., 1977), and in the hostile media phenomenon, where groups attribute bias (favoring the other side) to neutrally presented media content (Vallone et al., 1985). By contrast, motivated reasoning refers to cognitive processes that help us reach desired conclusions. “Directional goals […] affect people’s attitudes, beliefs, and inferential strategies in a variety of domains” (Kunda, 1990, p. 493). These processes may rest on the belief that one’s views, and those of one’s group, are particularly objective. In turn, the “biased assimilation” (Greitemeyer et al., 2009, p. 23) of new information in ways consistent with our beliefs buttresses groups’ resources for collective action in times of conflict.

### Static Objects and Projects in Intergroup Relations Research

Despite the underlying focus on conflict, very few studies have included research designs that consider the specific localized *content* involved in such universal psychological processes, such as actual societal beliefs about the conflict or the outgroup (Bar-Tal and Halperin, 2011, p. 229). This means that most intergroup research to date has not tapped into the historicity of conflict and how groups perceive relevant objects and the goals of their outgroup. Researchers often focus on similar objectifications of diverse phenomena in various domains. For instance, a social object like Islam may be linked to Moroccans in the Netherlands (Dekker and Van der Noll, 2012, p. 115), Turks in Greece (Sakellariou, 2017) and Libyans in Malta (cf. Buhagiar et al., 2018), despite the fact that the national groups involved are widely disparate in various characteristics. Consequently, Helbling (2012, p. 5) concludes that whether Islamophobia relates to Muslims or immigrants from the Arab world is an open question (Helbling, 2012, p. 5), especially since attitudes toward the latter tend to be worse than toward Muslims *per se* (Bleich and Maxwell, 2012, p. 45). A recent media analysis found striking similarities across six European countries (the United Kingdom, France, Malta, Italy, Romania, and Greece) in representations of Islam (Buhagiar et al., 2020). Such representations are anchored in different objectifications and construals of Muslims and Islam, and as such, these differences may be key to understanding changing intergroup relations over time. Similarly, Muslim communities re-negotiate representations of themselves and of intergroup relations with non-Muslims as well (Sartawi and Sammut, 2012). Our point here is that representations of the object ‘Islam’ must be operationalized in ways that make sense for the communities being studied, as opposed to glossing over its different objectifications across contexts.

As with objects, projects advanced by majority and minority groups cannot be operationalized in ways that ignore contexts and collective beliefs (cf. Bar-Tal and Halperin, 2011, p. 229), and that result in different construals by the parties concerned. Bar-Tal’s (2011, p. 1) step-wise definition of conflict above posits perception as a phenomenon *prior* to action. There has therefore been a focus on “perceptions in conflict” (Fisher and Kelman, 2011, p. 61), conceptualized as *preceding* the projects themselves. At best, such research focuses on “perceptual processes that can account for the impact of subjective factors on conflict escalation and perpetuation: the formation of enemy and self-images, and the resistance of these images to contradictory information” (Fisher and Kelman, 2011, p. 64). However, if group concerns do reflect motivated social cognition (Kruglanski, 1996; Krochik and Jost, 2011, p. 147), then questions of content must be tackled directly in research design, to study how different projects are served precisely by such cognitions. That is, how does the tailored re-presentation of objects enable “freezing” (Kruglanski, 2004, p. 14) on one specific project as opposed to another? For example, why do Jewish stereotypes of Arabs exhibit themes of primitive behavior (Bar-Tal and Teichman, 2005), and which project do such stereotypes advance: a one-state solution, a two-state solution or some other project (cf. McDonald et al., 2018)? Similarly, majority group members in the West tend to favor assimilation or colorblindness over multiculturalism (Verkuyten, 2014); nonetheless, “multiculturalism rather than colorblindness can be reassuring for high majority group identifiers” among Hindu majority members in Mauritius (Ng Tseung-Wong and Verkuyten, 2018). Such findings may seem anomalous, and a common practice is thus to include variables such as national contexts as moderating variables (see Guimond et al., 2014). However, we argue that contextualized projects are key to understanding such apparent anomalies. Another example concerns research on acculturation strategies (e.g., migrant integration, assimilation, etc.) and wellbeing in Canada (Berry and Hou, 2016). Here, generalized operationalizations of acculturation strategies and life satisfaction were used, even though construals of integrationist projects may vary widely across migrants. Yet, if groups construe projects differently and attribute beliefs to their outgroup in conflictual relations, then such construals are vital to understand intergroup relations, and should feature in variable operationalization. In summary, the above literature illustrates a key problem concerning the assumption of unchanging representations of objects and projects, whose understanding is deemed as being interchangeable among different groups.

### Objects Re-presented Without Projects

Much like mainstream research, SRT has sought to understand how certain objects are represented differently by different groups, with little regard for the projects being advanced. As we detail hereunder, such research has pursued an implicit object-oriented formula, which determines the focus of study as: the social representations of X [object], held by Y [group], in C [context]. This is the case in Moscovici’s (1961/2008) classical study *Psychanalyse*, where the object of psychoanalysis was studied in the French public as it is re-presented by three distinct groups: Catholics, Communists, and Liberals. Moscovici’s study was concerned with social representations *of* psychoanalysis (Object X), *by* Catholics/Communists/Liberals (Group Y), *in* 20th century France (Context C). Moscovici’s original formulation compares the content of three somewhat dissimilar social representations of psychoanalysis circulating in the same French public at one point in time. This seems to suggest that the object is represented differently by the three distinct groups in the French public. The naïve citizen is thus faced with three distinct social representations of psychoanalysis, as though psychoanalysis were a different object to one group than to another. Here, one should note that the preposition ‘of’ does not imply actual existence of the representation itself outside of discourse (cf. Potter, 2019), separately from the object; the object is simply “the *inside-view* or folk-expression for what a specific representation stands” (Wagner, 1996, p. 115). Nonetheless, this formula misses an understanding of why psychoanalysis is re-presented the way that it is by a certain group. Possibly, a group (e.g., Catholics) may re-present the object (i.e., psychoanalysis) in reaction to some other interested group’s representation of the object, as something that runs counter to the original group’s project (e.g., taking one’s tribulations to the psychoanalyst rather than the priest could be antithetical to the Catholic project). Therefore, whilst intergroup dynamics are implicit in Moscovici’s work, they were not methodologically exploited.

Moscovici’s (1961/2008) study was conducted in post-World War II France, where strong Catholic and Communist bases fought for hegemony in a political atmosphere influenced by the cold war (Duveen, 2008, p. xv). Psychoanalysis became an object through which such ideological battles were fought (Duveen, 2008, p. xv). However, the hypotheses formulated are almost exclusively dedicated to understanding the terms/themes used to discuss psychoanalysis (e.g., Moscovici, 1961/2008, p. xxxvi). That is, the focus was squarely on the object, with analytical considerations of action and intergroup relations being secondary. This meant that the study effectively remained silent on how groups can renegotiate their appraisals of objects in line with their projects and contextual demands *over time*. In fact, Moscovici asks how it is that, years later, one observes an improved relationship between Psychoanalysis and Communists (Moscovici, 1961/2008, p. 343), driven by various events including scholarly developments bridging both paradigms (p. 345). This blind spot may be the result of a synchronic focus on ‘social representations of Object X,’ as opposed to a longitudinal focus on ‘social re-presentation *for* Project P.’ Beyond the assumption of unchanging objects and projects highlighted above, this concern constitutes a second problem: that of studying re-presented objects without analyzing which projects they serve. We argue that a more nuanced appreciation of the relationship between collective action and re-presentation can redress this problem in intergroup relations research.

## Collective Action and Joint Intentionality

The necessity of project-focused research can be highlighted by making recourse to a central distinction between “representations-of” and “representations-for”: the former emphasize what is being re-presented whereas the latter highlight what it is being re-presented *for* (Millikan, 1989, 1995; Franks, 2011, p. 130). This distinction is best explained with reference to Franks’ (2011, p. 130) example conceptualizing retinal evolution as being driven by what the motor cortex needs visual representations of the world *for*. Foregrounding visual representations that are simply *of* the world leads to questions concerning the degree of representational accuracy needed for proper functioning. Contrarily, “representations-for” relegate issues concerning the accuracy of visual (or other) representations to a secondary role, and most importantly, fulfill the requirements of the “representation consumer” (e.g., the motor cortex) (Franks, 2011, p. 130). Applying this distinction to SRT, social “representations-of” exemplify the problems faced by studies such as Moscovici’s (1961/2008), whilst “representations-for” emphasize the primacy of joint intentionality and collective action as ultimate ends. We thus propose that studying social representations as a collective property (see Harré, 1984) of groups makes possible an understanding of social representations as enablers of joint intentionality and action.

Joint intentions are ones where a single agent would not suffice for their existence. Such intentions always involve more than simply a collocation of individuals (Franks, 2011, p. 42). For example, the intention to have dinner with friends is contingent upon its being shared by two or more people (Gilbert, 1989; Tomasello and Carpenter, 2007). To link joint intentions with SRT, social representations should be understood as collective – rather than distributive – properties of groups (Harré, 1984). Distributive properties are those attributable to individual members of a group: for example, the weight of an army is a distributive feature, as all members of an army weigh a certain amount and contribute to the overall weight. In contrast, collective properties are not attributable to individual members: for example, the tactical formation of an infantry in battle is not attributable to individual soldiers, but rather to the collective itself. Given that social representations are collective phenomena in this sense, then individual members can jointly position themselves *for* or *against* a representation because both promoters and opponents of a representation can access it (see Sammut, 2015).

Another feature of social re-presentation that accords with this view is its tolerance for contradiction. Contrasting representations may be discursively elaborated by the same group in the service of a common goal (Potter and Litton, 1985). Whether one identifies coherent notions or else emphasizes contradictions in speech depends on one’s motivation (cf. Billig, 1987, p. 263). Coherence and contradiction are “competing strategies” (p. 254) for influencing one’s audience. For instance, Rose et al. (1995) note how in Nazi Germany, Jewish migrants were seen as being both ardent communists and staunch capitalists. Here, delineated common understandings of Object X become a moot point. What is necessary for social action is neither an exhaustive elaboration of social representations, nor representational consistency (Fraser, 1994), but adequate and sufficient re-presentation on which action can be based and outcomes pursued (see Roqueplo, 1990; as cited in Lahlou, 2015, p. 201). For example, ‘Jews as communists’ and ‘Jews as capitalists’ could both suffice to promote anti-Semitic projects. Moreover, it is rarely possible to decouple descriptive features of representations from prescriptive ones (Fraser and Gaskell, 1990; Harré, 1998, p. 132; Moscovici, 2000, p. 21). This becomes evident when one considers the range of qualitative methods employed in SRT (e.g., interviews, focus-groups, etc.; Wagner et al., 1999) and the results obtained. Presumably, participants contribute to such studies principally, though by no means exclusively, by describing their views on social objects rather than continuously and explicitly prescribing courses of action entailing the object. Nevertheless, a common normative thread is usually discernible in the results of such studies, that is, respondents would be *for* and *against* some course of action.

This understanding of ‘re-presentation *for*’ prioritizes the systemic and functional aspects of social re-presentation, and carries two key implications. Firstly, the functionality of representations takes primacy over description. Representations are primarily pragmatic (see Fiske, 1992) and only secondarily concerned with semantic truth (Wagner and Hayes, 2005). Therefore, social representations are real because people act accordingly (Bauer and Gaskell, 2008), their “as if” nature being dependent on practical solutions (Wagner and Hayes, 2005). Consequently, the “stickiness” of representational content (that is, why some content retains a certain currency; Breakwell, 2014, p. 126) is better explained with recourse to action trajectories and contexts exterior to the object (cf. Bauer, 2015, p. 60). This implication supports the inclusion of joint projects as a third analytical component (i.e., together with ‘groups’ and ‘representations’) in research designs.

Secondly, joint projects (Bauer and Gaskell, 2008) need not be made explicit to be actuated, and individuals need not act explicitly to actualize representations (Wagner, 2015). If the prescriptive side to re-presentation were constantly evident, its strength would diminish accordingly, as the co-constructive element inherent in re-presentation would be tainted at every step by attributions of utilitarian intent. Consider the bilateral straw man arguments partaking in alternative representations within intergroup contexts. Alternative representations are representations “*of a potentially competing representation from within a social representation*” (Gillespie, 2008, p. 380). Here, outgroup representations are invoked in a purposefully simplistic manner, only to be easily dismissed in favor of the ingroup’s alternative. These representations contain multiple implications (Moscovici, 1994) concerning outgroups. They serve to coordinate action vis-à-vis that outgroup by virtue of their prescriptive element being implicit in their descriptive elaboration. For example, in *Psychanalyse*, when Communists alternatively re-present psychoanalysis as “American psychoanalysis,” this frames psychoanalysis politically, putting it in direct opposition with Communist goals (Gillespie, 2008). This highlights the salience of group membership, and its implication for intergroup relations and the policing of group boundaries. Similarly, outside of SRT, research by Peery and Bodenhausen (2008) and Chen et al. (2018) in the US showed a *minority bias* where multiracial participants were more likely to be categorized as ‘black’ or ‘not white.’ However, the samples barely included any black participants. Following the logic of alternative re-presentation, it would be interesting to see whether black participants would exhibit similar biases, or else re-present multiracial faces and exhibit other biases in accordance with different (implicit) projects instead.

### Collective Action: SRT and Social Identity

Beyond SRT, a dominant approach that has theorized collective action is that of social identity theory (SIT; Tajfel and Turner, 1979) and related models (e.g., van Zomeren et al., 2008). According to SIT, individuals want positive self-esteem and thus engage in strategies aimed at enhancing/maintaining their self-esteem. Such strategies include leaving a group and joining another in order to forge a different social identity; direct competition in pursuit of increased status; and creative re-appraisals of one’s positive characteristics that help redefine one’s identity in positive terms (Tajfel and Turner, 1979). van Zomeren et al. (2008) developed the social identity model of collective action (SIMCA) by incorporating identity, perceived injustice and perceived efficacy as key variables in determining whether people are more likely to engage in collective action. According to SIMCA, identity predicts collective action in three ways. It does so directly, indirectly by underpinning shared experiences of injustice, and indirectly because the stronger one’s identification with a group, the more efficacious one feels in bringing about social change. In SIMCA, social identity predicts perceptions of both injustice and efficacy (van Zomeren et al., 2008). More recent work has incorporated identity, efficacy, emotion and morality as key predictors in the model (van Zomeren, 2013) and started addressing cultural variance (van Zomeren, 2019).

van Zomeren 2013, p. 385) notes that SIMCA “is not grounded in a larger theory about human functioning” and should be embedded into a more general theory. Our contribution also highlights the opposite: SIMCA can benefit from including more specific project-related variables that clearly specify what courses of action individuals prefer over others. These should be in line with strategies (national, local, etc.) being discussed at the time, accessible by lay audiences. Revealingly, van Zomeren et al. (2018) argue that “identity content remains neglected in research on collective action, notwithstanding the lip service paid to its presumed importance” (van Zomeren et al., 2018, p. 127). They note how it might not be categories themselves that motivate people for action, but the *content* of such categories (van Zomeren et al., 2008, 2018). The authors present examples, such as Becker and Wagner’s (2009) observation that identification with the category ‘women’ can mean identification both with feminist or traditionalist women, carrying substantial implications for research outcomes (e.g., which gender roles one supports). The fact that identity content is “assumed but not assessed, and interpreted but not actually tested” (van Zomeren et al., 2018, p. 127) mirrors our concern with operationalizations of collective action itself. It is perhaps not collective action itself (as a general process and undifferentiated outcome variable) that is intended for by members of a group, so much as the fulfillment of contextualized content-based projects.

### Joint Projects

Action has been problematized by different traditions of research within SRT and remains of concern (see Batel and Castro, 2018; Potter, 2019). These conceptualizations can advance our understanding of collective action. Castro and Batel (2008) argue that there are three ways of viewing action and re-presentation within SRT. Firstly, the constitutivist view sees action as being part and parcel of re-presentation (e.g., Wagner, 1996, 2015); for example, the act of re-presenting Muslims as terrorists (e.g., Buhagiar et al., 2020) simultaneously achieves things in the world. Secondly, the functional view conceptualizes re-presentation as capable of serving certain functions and actuating things; this accords with the view that “social representations are often only apparent in action” (Howarth, 2006, p. 72). Here, the same representation *promotes* courses of action over others but remains separate from tangible courses of action (such as supporting ethno-nationalist causes against Muslims). Thirdly, the creative view emphasizes how re-presentation creates new possibilities of behaving (Castro and Batel, 2008). For example, groups who re-present Muslims as terrorists might commit ‘novel’ acts such as avoiding travel to Muslim-majority countries altogether. These proposals shed light on different aspects of re-presentation; they need not be mutually exclusive. All three become meaningful when understood in view of joint projects (Bauer and Gaskell, 1999), which, we contend, offer a viable and solid basis for the proposed reformulation. It is “future-making” and ideological activity (Jovchelovitch, 2007, p. 87) that make evident “*the power* [of social representations] *to shape mutual expectations within a collective* in such a manner as to enable or impede coordinated actions directed toward a given purpose” (Elcheroth et al., 2011, p. 745; see Howarth et al., 2014).

Bauer and Gaskell (1999, 2008) propose a “toblerone model” of social re-presentation that features the notion of joint projects. The toblerone model explains how subject-subject relations temporally re-present objects to advance joint projects (Bauer and Gaskell, 1999). An object is here defined broadly as either an abstract notion or a concrete entity. Projects are the pragmatic contexts within which joint sense-making and action occur in a collective; they link subjects together within a common trajectory (Bauer and Gaskell, 1999, 2008; Bauer, 2015). The toblerone model defines re-presentation as a “time-gestalt of ‘inter-objectivity’” (Bauer and Gaskell, 1999, p. 171), framing action and serving identity and memory functions within a community (Bauer, 2015). In this model, social representations are trebly construed as incorporating representations of object, project and subjects (Bauer and Gaskell, 1999); and subjects within re-presentation are always a collective in the first-person plural (Bauer and Gaskell, 2008), rather than atomized individuals (see Harré and Secord, 1972; Wagner, 1996, 2015; Marková, 2000). A project is necessarily *joint*; it does not simply mobilize singular subjects based on their separate intentions. This mobilization is teleological, projected toward desired states of affairs that are inherent in there being a “not-yet” and a “future-for-us” (Bauer and Gaskell, 2008, p. 343).

Later developments of the toblerone model included multiple “toblerones” of different sizes, signifying power relations between dominant and non-dominant groups (Bauer and Gaskell, 2008). This consideration of power relations introduced an explicit focus on intergroup dynamics in SRT. The notion of joint projects allows for “a more explicit conceptualization of action and interaction” (Foster, 2011, p. 23.2). Under this view, re-presentation is always *for* (Millikan, 1989, 1995; Franks, 2011, p. 130) some course of action over another, evincing the claim that “theories on SR [social representations] are also fundamentally theories of social *power*” (Elcheroth et al., 2011, p. 747). This view incorporates a more nuanced sensitivity for action, and can shed light on dynamics of mobilization within and across social movements. Nevertheless, social representations research does not necessarily fulfill Bauer and Gaskell’s (1999, 2008) aspirations, as detailed above. Social representations emerge, reproduce and are challenged by means of “asymmetrical intergroup communication and influence” (Staerklé et al., 2011, p. 759). Yet, despite the relevance of conflict in SRT (Elcheroth et al., 2011; Staerklé et al., 2011), intergroup dimensions are not studied in terms of clashing projects. We now proceed to synthesize the above review and present both the object-oriented formula and our action-oriented reformulation in more detail.

## ‘Social Representations Of…’: Object-Oriented Research

We term *object-oriented* any view that studies social representations as being *of* Object X, held *by* Group Y, and found *in* Context C. Symptomatic of this view are attempts at fleshing out what the characteristics of social representations of Object X might be, without studying why they are such. This formula can thus portray social representations as static and unitary entities (see Potter and Litton, 1985). The quintessential example of a strong object-orientation involves operationalizing social representations as discrete variables (see Marková, 2000). This promotes an exclusively distributive view (Harré, 1984) of social representations, which are to be “found” among a determinable number of individuals, but absent in others. Under this view, social representations are social simply because they are present in more than one specimen. Object-oriented views also typify much research where social representations are understood as being mutually *constitutive* of social groups, even if no case is made for treating representations as discrete variables. Essentially, such research fits the following formula:

Social representations SR^n^ of Object X, by Group Y, in Context C

This formula excludes considerations of (a) the projects being advanced, and (b) how different groups re-present objects and the projects of their outgroups in ways that serve their ingroup’s project.

### Object-Oriented Research in SRT

Examples of object-oriented research abound in the literature. We review work concerning the social representations of human rights (Spini and Doise, 1998; Doise et al., 1999; Doise, 2002), and more recent examples concerning social representations of Latin American history (Brasil and Cabecinhas, 2017) and of secularism (Troian et al., 2018). We also present illustrative examples of mainstream object-oriented research concerning perceptions in intergroup relations (Cvetkovska et al., 2020).

#### Social Representations of Human Rights

Spini and Doise (1998) hypothesize and show that the social representations of personal and governmental involvement in human rights have both an abstract and an applied dimension. The authors state that social representations should be situated “in the social history of individuals and groups” (Spini and Doise, 1998, p. 607). Nonetheless, little to no consideration is given as to what the representations of human rights are *for*. Despite various opportunities for discussing potentially contrasting projects and their influence on the social re-presentation of human rights among different collectives (e.g., Catholics, Protestants, etc., who were present in the sample), the authors favor a research question focusing on an amorphous “people” and their views. The authors are clearly concerned with the following formulation:

Social representations SR^n^ of Object X [human rights], by Group Y [psychology/sociology/law students], in Context C [Geneva]

Revealingly, Doise (2002, p. 143–144) notes that when human rights issues are contextualized, social representations change accordingly. For example, human rights violations can be tolerated when the person in question occupies a marginal social position. The more an issue is contextualized, the more projects rise to the surface. However, Doise (2002) discusses such “inconsistencies” (p. 145) in terms of rationalizations and dissonance, instead of adopting a more ecological view of reasoning (Goldstein and Gigerenzer, 2002) across different project-driven collectives. Our proposed reformulation here recommends (a) a research design focused on projects in which human rights are relevant. The authors note that human rights are sometimes regarded as Western exports based on Enlightenment ideas, and that “the more such ideas prevail in a culture, the more positive attitudes should be toward [human rights]” (Doise et al., 1999, p. 5). The exportation of human rights discourse to other countries could have constituted the project under investigation; it would have been fruitful to see whether, for example, universalist values (which are explored in the study) serve to promote foreign interference. Secondly, the employment of representations for or against the specified projects could have been studied using (b) outcome measures that reflected such projects, and (c) samples consisting of salient groups (relevant to the research question) sharing a meaningful intergroup relation.

#### Social Representations of Latin American History

In contrast to Spini and Doise (1998); Brasil and Cabecinhas (2017), in their study on social representations of Latin American history, explicitly note how collective memory serves “to mobilize the sense of belonging to the ingroup, contributing to the tendency of group members to engage in collective projects due to their shared belonging” (Brasil and Cabecinhas, 2017, p. 540). The authors conducted a survey with Chilean, Brazilian and Mexican students, asking them to list events/personalities they deemed as being the most historically important, and to indicate the influence they thought such events/personalities had on Latin American history. The students emphasized conflict-related and political events, events related to colonialism, and country-specific events. The latter finding was taken as evidence for a sociocentric bias. The authors further note that “social representations of history may serve as a tool to segregate and reinforce stereotypes” (Brasil and Cabecinhas, 2017, p. 551). Nonetheless, this study essentially looked at:

Social representations SR^n^ of Object X [Latin American history], by Group Y [Chilean/Brazilian/Mexican students], in Context C [online context]

Despite mention of projects and social representations of history acting “as a tool” (Brasil and Cabecinhas, 2017, p. 551), it is not clear against or for whom this tool is used. This is even more pressing given that collective memory “is one of the major epistemic bases for delineating courses of action and motivating mobilization” (Bar-Tal, 2014, p. 5.4). Here, it would have been suitable to (a) focus on intergroup reconciliation between colonizers and the colonized, which is at times alluded to in the manuscript, or any other relevant project. The authors argue that certain representations of history “could also be a way of blaming the Other (in this case, the countries that were once the colonizers) for the difficulties that the individuals from this region face today” (Brasil and Cabecinhas, 2017, p. 552). Thus, there was the possibility of operationalizing a key project sensible to the Latin American context vis-à-vis past colonizers. How colonizers and the colonized alternatively re-present each other’s project is also key to understanding social representations “as a tool.” Secondly, (b) this study could have analyzed the uses of history by incorporating outcome measures aimed at getting both groups’ views, and their views of each other’s views on the project in question. Thirdly, (c) sample constitution could have reflected both groups in question.

#### Social Representations of Secularism

Troian et al. (2018, p. 96) test whether social representations of secularism serve as a “legitimizing myth” promoting prejudice toward North African minorities, and whether “new secularism” (i.e., the view that persons should be laic and religious symbols excluded from public life) mediates the relationship between social dominance orientation (SDO; Sidanius and Pratto, 2001) and prejudice. This bears “important stakes in the present political and societal situation (i.e., controversies surrounding the ‘*burkini*’ and the following discriminatory practices of civil servants toward minorities)” (Troian et al., 2018, p. 102). The authors distributed a survey including a Secularism scale, an SDO scale and a Generalized Prejudice scale (Dambrun and Guimond, 2001). This study thus related to:

Social representations SR^n^ of Object X [Secularism], by Group Y [French people], in Context C [France]

To account for motivated reasoning, the research design could have benefitted (a) by incorporating explicit projects relating to North Africans in France: do native French participants (without North African descent) favor migrant assimilation, repatriation, integration, or other acculturation strategies? Instead of static depictions of “representations of secularism,” the strategies for which such representations might be employed could have been a fruitful addition to the tested mediation model, where (b) the outcome measure becomes that of a *contextualized* project rather than *generalized* prejudice. Moreover, looking at what North Africans think about such projects, and how they in turn re-present secularism for/against them is also recommended. Finally, (c) asking both groups about their views of each other’s projects would have captured the systemic element endemic to social re-presentation. Collecting data from “civil servants [and] minorities” (Troian et al., 2018, p. 102) is equally pertinent.

### Mainstream Object-Oriented Research: Toleration and Group Identification

Cvetkovska et al. (2020) recently published an interesting study concerning relations between toleration, well-being, and group identification among minorities in the Netherlands. Toleration was associated with higher well-being through higher national identification. Interestingly, statistical associations between well-being, group identification and *toleration* tended to fall between the associations of such measures with either *discrimination* or *acceptance*. Viewing acceptance (rather than toleration or discrimination) as the most adequate descriptor of ingroup treatment was associated with higher national identification. Contrastingly, discrimination was associated with stronger ethnic identification. The authors argue that “this suggests that toleration gives less impetus to use one’s ethnic group as a resource for improving one’s well-being, compared to being overtly discriminated against” (Cvetkovska et al., 2020, p. 166). However, an alternative explanation could be that high ethnic identification (group) pushes group members to re-present perceived intergroup relations (object) as something else other than a situation involving toleration (e.g., as one involving discrimination). The project advanced by those with higher ethnic identification could be different than that advanced by those with lower ethnic identification or higher national identification. Hence, minorities may shift alliances between ethnic and national groups given the project they seek to advance. More alignment with the national group means that a situation of more intergroup acceptance is perceived (object), advancing Project X (e.g., the project of Laïcité in France, for secularism at a national level). Similarly, higher alignment with one’s ethnic group means that the same situation is perceived as involving more discrimination (object), thus advancing Project Y (e.g., the project of recognizing and having a legitimate place for Muslim practices). The authors instead argue for a reparative view of identification, where perceptions of ingroup treatment somewhat took precedence over group identification: “it may be that one cannot help but feel negative emotions when perceiving one’s in-group to be discriminated against. Ethnic group identification may restore positive feelings of self-esteem and connection” (Cvetkovska et al., 2020, p. 170). Essentially, this research fits the following formula:

Social representations SR^n^ of Object X [toleration/ discrimination/acceptance], by Group Y [Turkish/Moroccan/ Antillean/Surinamese minorities], in Context C [Netherlands]

## An Action-Oriented Reformulation

As highlighted above, motivated social cognition (Kunda, 1990; Kruglanski, 1996; Molden and Higgins, 2005) is key to understanding intergroup relations. It follows that a primary focus on objects or perceptions themselves can limit the capacity to account for disparate findings across domains, even if causal explanations demonstrate the functionality of attitudes/perceptions. Our proposal is applicable to various domains where perceptions in intergroup relations have an impact on behavioral outcomes. A statement by Adelman et al. (2019) can be used to illustrate this point: “motivated reasoning may not only influence when we assign [judgments to groups], but also how it shapes the perception of the nature of the group in question to be consistent with the judgment [itself]” (Adelman et al., 2019, p. 37). This begs the following question: why isn’t the whole reasoning process described by Adelman et al. (2019) itself conceptualized as motivated reasoning, with its motivating cause being an underlying project (which would have to be tested experimentally)? In other words, to what end is such reasoning motivated? Having conceptualized social re-presentation as being *for* joint projects, we now propose a reformulation that (a) is action-oriented, and (b) furthers the study of joint projects and alternative re-presentation (Gillespie, 2008) in intergroup scenarios.

### ‘Social Re-presentation for…’

In applying the distinction between “representations-of” and “representations-for” to *social* re-presentation, the object-oriented view (“Social representations SR^n^ of Object X, by Group Y, in Context C”) can be reformulated as follows:

Social re-presentation SR for Project P, of/as Object O, by Group G_1_, in Context C…according to Group G_x__…__n_

This systemic formula (a) foregrounds social re-presentation as process, serving collective functions (“Social re-presentation SR”); (b) incorporates action (“for Project P”); (c) expands upon variants of re-presentation, allowing for both realist and social constructionist emphases (“of/as Object O”); (d) redefines groups as joint subject-subject relations on a collective level (“Group G_1_”) (Bauer and Gaskell, 1999); and (e) allows for the alternative re-presentation of project trajectories by multiple groups relevant to Context C (“according to Group G_x__…__n_”). The point we wish to make here is that the motivational basis for transforming certain ideas into social representations can only be substantiated with reference to what representations are *for* (cf. Jahoda, 1988).

### ‘Alternative Re-presentation for…’

Social representations theory studies situations where groups intersect and resist each other, and knowledge encounters play out in public spheres (Bauer, 2015, p. 61; Jovchelovitch and Priego-Hernández, 2015, p. 167). It is divergent *practices* that make social representations visible (Bauer and Gaskell, 1999). Social groups and their projects are always vulnerable to alternative re-presentation by outgroups (Gillespie, 2008), and these in turn can be resisted or engaged with in various ways (Jovchelovitch and Priego-Hernández, 2015, p. 173). The present reformulation incorporates social representations alongside alternative representations that may interrelate to and fro across social groups longitudinally. It enables research of a group’s reactions to the alternative representations leveled at it by its outgroup(s), and vice versa. Expanding upon Bauer and Gaskell’s (1999) systemic conception, the reformulation addresses: “how in the object, the project of the subjects is represented; or how in the subjects the object appears in relation to a project; or how the project links the subjects and the object” (Bauer and Gaskell, 1999, p. 168), as per the following formula:

SR for P_x__…__n_, as a function of SR_1_, AR^2^, AR_1_^n^, SR_2_, AR^1^, AR_2_^n^ and any other SR_n_ and AR_n_ relevant to the context

Thus, if one were to focus on “how in the object, the project of the subjects is represented” (Bauer and Gaskell, 1999, p. 168), one can substitute P_1_ for P_x__…__n_ to consider the formula specifically from the viewpoint of G_1_. Here, social re-presentation for P_1_ by G_1_ (SR for P_x__…__n_) is a function of (a) how G_1_ socially re-presents the Object/s in question (SR_1_); (b) how G_1_ alternatively re-presents the Project of its outgroup (AR^2^) and that of other relevant groups (AR_1_^n^); (c) how the outgroup socially re-presents the Object/s in question (SR_2_); (d) how the outgroup alternatively re-presents the Project of G_1_ (AR^1^) and that of other relevant groups (AR_2_^n^); and (e) so on for any other relevant groups in the same context (SR_n_ and AR_n_). In being advanced, P_1_ potentially incorporates or interrelates with other functionally relevant viewpoints in a systemic gestalt. Equally, if one were to focus on “how in the subjects the object appears in relation to a project; or how the project links the subjects and the object” (Bauer and Gaskell, 1999, p. 168), the formula can work without tying P_x__…__n_ to any specific group.

In line with the toblerone model (Bauer and Gaskell, 1999, 2008), the fuzzy conceptual nature of the formula components, particularly Object O, means that these can only be defined with reference to the project (P_x_) as it is embedded in subject-subject relations (G_x_). Contrary to object-oriented formulations, alternative re-presentation is conceptually linked to an outgroup’s Project in intergroup scenarios. Projects are questioned from without, and alternative representations of relevant objects follow suit. This mode of enquiry provides a neater solution to the question concerning how collectives use representations multifariously for specific ends (Jahoda, 1988). Accordingly, this systemic reformulation promotes researching the dynamism of collectives and their project/s over time, seeking to understand how collective projects shape and are in turn shaped by intergroup relations. We now proceed to present an illustrative study showing how an action-oriented approach improves intergroup relations research.

## Action-Oriented Research: ‘Social Re-Presentation for Migrant Integration’

We conducted research (Buhagiar et al., 2018; Sammut et al., 2018) concerning social re-presentation for migrant integration, using the above reformulation. Specifically, we looked at how the Maltese socially re-present the project of Arab integration in Malta. Interviews were conducted asking Maltese participants for their views concerning the *integration* of Arabs. Questioning was thus squarely focused on a project (integration) and the interviews engaged participants in argumentation. Participants were asked for their viewpoints, justifications, examples, qualifiers and exceptions to their central claims. We therefore studied “how in the subjects [Maltese] the object [Arabs] appears in relation to a project [integration]; or how the project [integration] links the subjects [Maltese] and the object [Arabs]” (Bauer and Gaskell, 1999, p. 168).

In essence, our study (Sammut et al., 2018) adopted an action-oriented empirical focus. The findings resulted in a wide array of pro- and anti-integrationist arguments, clustered around several themes. There was a predominance of anti-integrationist arguments, and whilst most argumentative themes traversed positive, ambivalent and negative stances toward integration, no positive religious arguments for Arab integration were made (Sammut et al., 2018). The action-oriented focus thus helped, crucially, “to identify instances where certain semiotic resources might be absent in certain social representations” (Sammut et al., 2018, p. 8), perhaps because such content would directly oppose the project being advanced. Among the arguments concerning integration, we highlighted those converging around socio-political themes. For instance, Arabs were re-presented as longstanding trade partners, serving to promote the benefits of Arab integration in Malta (Sammut et al., 2018). Here, the emphasis was on the economic benefits of integration and the possibility of co-existence, as evidenced by a history of Arab-Maltese relations. Accordingly, this social re-presentation comes together in view of an integrationist project, and can be formulated as follows:

Social re-presentation SR [Arabs as trade partners] for Project P [integration], of/as Object O [Arabs], by Group G_1_ [Maltese], in Context C [Malta]…according to Group G_x__…__n_ [Maltese]

Moreover, as per our second proposed formula, an action-oriented approach allows for further consideration of alternative re-presentation and intergroup dynamics, both of which can modify the social re-presentation processes under study. This research is being developed by posing the same questions to Arabs, to investigate their social re-presentation of the integrationist project. Future research will also address alternative re-presentation by asking Maltese respondents what they think Arabs make of integration, and Arab respondents what they think the Maltese make of integration. This overarching programmatic endeavor can be expressed as follows:

SR for P_integration_, as a function of SR_M_, AR^A^, AR_M_^n^, SR_A_, AR^M^, AR_A_^n^ and any other SR_n_ and AR_n_ relevant to the context

Here, the integrationist project (P_integration_) is a function of (a) how the Maltese socially re-present Arabs in Malta (SR_M_) and alternatively re-present their project (AR^A^) and that of other relevant groups (AR_M_^n^), and (b) how Arabs socially re-present themselves in Malta (SR_A_) and alternatively re-present the project of the Maltese (AR^M^) and that of other relevant groups (AR_A_^n^). This aligns with Tajfel’s (1984, p. 696) view of social representations as a collective background mobilizing intergroup relations; and provides a way for studying changes in social representations that unfold as a function of minority/majority relations (Marková, 2008).

This research program studies how perpetual re-articulation of others’ projects can in turn serve one’s own. Had our study adopted an object-oriented design, it would have chiefly focused on social representations of ‘Arabs’ amongst ‘the Maltese’ in Malta. The research would have tied itself closely to the Object, whereas the fuzzy nature of the Object might be precisely what is exploited in intergroup scenarios in order to legitimize one’s project over others’ (see Table 1). For instance, whether interviewees speak about ‘Arabs,’ ‘Arabs in Malta,’ ‘Arabs in Europe,’ ‘Arab-Maltese relations’ or even specific Arab nationalities, can differ across groups advancing contrasting projects. Were the focus to be on the object, the interviewer and interviewee might have still reached an understanding concerning the topic of discussion. However, asking about a project achieved this and even more. For instance, the focus on joint projects also allowed for an abductive analysis that showed how arguments from cultural essentialism advance anti-integrationist projects (Buhagiar et al., 2018). This argumentation strategy involved reducing Arabs to their cultural dimension, which was seen as having a determinist influence on Arabs in a persistent manner over time and as opposing Maltese culture (Buhagiar et al., 2018).

**TABLE 1:** Differences between the object-oriented and the action-oriented approach to social psychological research.

Feature	Empirical orientation	
	Object-oriented	Action-oriented
Object	Representations of the object are foregrounded in the research design	Representations of the object are not foregrounded in the research design
Project	Projects are treated *post hoc*, if at all	Projects are foregrounded in the research design
Action	Action is treated *post hoc*, if at all	Action considerations inform the research design
Analytical frame	Lack of analytical third factor: group-representation correspondence	Inclusion of analytical third factor: group-representation-action triad
Pluralities	Prone to distributive view; may or may not be concerned with collective pluralities	Antithetical to distributive view; necessarily concerned with collective pluralities
Stickiness	Unclear what makes representations stick (infinite regress)	Stickiness of representations explained with recourse to projects
Social representations	Substantive descriptions; content determinable in cross-sectional research (‘of’)	Functionalist descriptions; content only determinable vis-à-vis projects longitudinally (‘for’)
Alternative representations	May feature as a matter of happenstance	Are central; reflect the systemic nature of re-presentation
Intergroup relations	Main focus on comparisons between group representations; intergroup relations treated *post hoc*, if at all	Main focus on interaction between group projects; intergroup relations central
Social influence	Non-systemic	Systemic

*This table compares the object-oriented approach with the action-oriented approach on 10 key points.*

Additionally, asking about the ‘integration of Arabs’ cut to the chase, allowing the interviewees themselves to elaborate the object from whichever angle they preferred. This shed light on which emphases advance specific projects over others. Mutual understanding between interviewer and interviewee is not compromised given that the project in question is signified by a lay term or else by one that has penetrated the consensual sphere. ‘Integration’ makes lay sense in the present socio-political climate of Malta, having infiltrated common parlance. By asking about integration, participants’ views were expressed more cogently, with representations of the object following suit: in advancing integration, Arabs were construed as an asset in Maltese-Arab relations (Sammut et al., 2018); in resisting integration, Arabs were culturally essentialized (Buhagiar et al., 2018), and so on.

## Discussion

In this paper, we revised two formulas for intergroup relations research. The first formula directed intergroup relations research from focusing on ‘social representations of’ to focusing on ‘social re-presentation for,’ foregrounding functionality. In turn, the second formula elaborated upon the first by formulating the project as a function of intergroup relations, prioritizing the systemic nature of social/alternative re-presentation. In essence, object-oriented and action-oriented approaches differ in their understanding of 10 key points: the object; the project; action; the analytical frame employed; the nature of pluralities; representational stickiness; intergroup relations; conceptions of social representations; conceptions of alternative representations; and the nature of social influence (see Table 1).

As detailed above, the difficulties of an object-orientation are threefold: (a) an omission of concerns with varying representations of objects and projects in mainstream research; (b) ambiguity or neglect concerning the collective nature of social re-presentation (Harré, 1984), specifically as pertaining to joint intentionality (Tomasello and Carpenter, 2007); and (c) an omission of considerations of the social/alternative re-presentation of joint projects (Bauer and Gaskell, 1999). We have argued that an action-oriented reformulation addresses these issues in ways amenable to improved empirical research. Action-oriented research better elucidates how the functions of representations change for groups over time–a poorly researched area of inquiry (Breakwell, 1993). The emerging view is one where social re-presentation binds coalitions for action. Coalitions re-present objects for joint purposes, in ways that accord with their surrounding sociocultural context (Sammut and Buhagiar, 2017), mobilizing subjects for joint action.

We also note that this view of re-presentation serves synthetic roles (cf. Parker, 1987; Bauer and Gaskell, 2008). It promotes a rapprochement between SRT and evolutionary social cognition on the one hand, and between SRT and discursive psychology (DP) on the other (see Batel and Castro, 2018). The link with evolutionary social cognition lies in a common functionalist basis, sharing the view that “thinking is for doing” (Fiske, 1992, p. 877). Whilst reproductive fitness signifies a distinct type of functionalism, it is wholly compatible with functionalist psychological approaches studying goals and motivated processes (Neuberg and Schaller, 2015, p. 9).

### SRT and Discursive Psychology

The link with DP is more nuanced. DP is already premised on the indispensability of action-oriented views of discourse (Heritage, 1984), where discourse is seen as having primarily practical consequences (Wetherell and Potter, 1988, p. 168). This paper builds on the convergences between SRT and DP (Batel and Castro, 2018) by incorporating functional concerns in the analysis. Under the action-oriented reformulation, re-presentational activity is conceptualized as furthering projects in line with ideological concerns. The focus on projects reconciles SRT with Billig’s notion of ideological dilemmas (Billig et al., 1988), where different projects might be constitutive of different ideological strands pulling collectives in contrasting representational trajectories. This can either result in conflicting ideological trajectories within the same collective project, or else in a situation involving conflicting projects altogether (cf. Billig et al., 1988). This constitutes a key point of convergence between SRT and DP, where ideology is already a central concern (cf. Billig, 1991; Batel and Castro, 2018). Moreover, we agree with Potter (2019) that analytic methods like thematic analysis are too coarse for appreciating the functions of language: the horizontal structure of most thematic maps does not highlight the causes being advanced by interlocutors. One strength of SRT was that “it highlighted the significance of representations for action” (Potter, 2019, p. 410). However, Potter argues that there are “confusions over *what* the representation is, *where* it is, and what its *role* is” (Potter, 2019, p. 411). We believe that our action-oriented reformulation provides the answer: re-presentation *is* the building block of discourse, *is found* wherever discourse is (i.e., “embedded in actions, sequences, and everyday and institutional practices”; Potter, 2019), and *has the role of* actuating joint projects, shared by members of a collective.

Moreover, as per our research above, researchers can map interlocutors’ discourse into claims, analyzing what these claims are *for*. Our approach adds the realization that the end goal of discourse ultimately lies outside of it: discourse is motivated and framed in line with joint projects. DP’s historical stance against cognitivism (see Edwards, 2012; Augoustinos, 2017) limits its possibilities for studying motivated reasoning. Yet, to understand why people are talking about, say, climate change in the way they are, the text itself cannot be the final litmus test.

### Recommendations for Future Research

Before concluding, we make a few recommendations for action-oriented social psychological research. These concern (a) how to frame research questions; (b) how to incorporate contextual factors in research; and (c) research design more broadly. It is recommended that research questions be as specific to the context as possible. Whereas universal cognitive processes are important (Bar-Tal and Halperin, 2011, p. 228), contextualized research questions/outcome measures approximate local projects more. General outcome measures can be more sensitive to social desirability biases (e.g., Campbell and Herman, 2015; van Niekerk and Verkuyten, 2018), further consolidating the need for context-specific measures. Research questions in intergroup relations should foreground the ways that outgroups alternatively re-present each other’s projects. Such questions can be reflected in measures such as: “What do you think [Outgroup X] thinks about [Project P]?”

This form of questioning only makes sense if contextual factors are incorporated in research. Qualitative work – whether naturalistic (Potter, 2019) or self-report (e.g., in-depth interviews) – can precede quantitative work and inform scale formation and survey composition, such that these then make sense for all groups being studied. This means revising the assumption of universalism and utilization of hybrid samples. A scale can be built based on qualitative data from groups in conflict and scaled such that it measures support for or against Project P. It can then be administered, asking both groups what they think, and what they think their outgroup thinks. This provides reliable and ecologically valid data. In cases where relevant projects are difficult to identify, open-ended questioning and subsequent analytical categorizations can help formulate outcome measures relevant to the context.

## Conclusion

The historicity intrinsic to social re-presentation (Villas Bôas, 2013) and social psychology more generally (Gergen, 1973; Billig, 2018; Wagoner and Brescó de Luna, 2018) validates the action-oriented formula, as this provides a skeleton for carrying out longitudinal research tracing joint projects (Sammut et al., 2012; Bauer, 2015, p. 57). Our reformulation shifts the research focus away from the automatic centrality of representational content, and looks at how dominant and subordinate groups re-define the meaning of contextual objects in view of their own motivated reasoning (Kunda, 1990; Molden and Higgins, 2005), and given their different resources (Staerklé et al., 2011). It is hoped that the proposed reformulation provides a way precisely to study this oft-neglected dynamic.

## Author Contributions

LB wrote the manuscript and GS provided critical revisions. Ideas and lines of argument were developed by both authors. Both authors provided approval for publication of the content.

## Conflict of Interest

The authors declare that the research was conducted in the absence of any commercial or financial relationships that could be construed as a potential conflict of interest.
